# Comparative study of physicochemical composition and microbial community of Khoormog, Chigee, and Airag, traditionally fermented dairy products from Xilin Gol in China

**DOI:** 10.1002/fsn3.2131

**Published:** 2021-01-19

**Authors:** Liang Guo, Weiliang Xu, Chundong Li, Yuansheng Guo, Yamei, Irbis Chagan

**Affiliations:** ^1^ Xilingol Vocational College Xilin Gol Institute of Bioengineering Xilin Gol Food Testing and Risk Assessment Center Xilinhot China; ^2^ Faculty of Life Science and Technology Kunming University of Science and Technology Kunming China

**Keywords:** Airag, Chigee, Khoormog, microbiology, nutrition

## Abstract

Due to their outstanding nutritional and functional properties, the traditionally fermented dairy products (TFDP) from camel, mare, and cow gained universal praise during their long history of production. In this study, the physicochemical composition and microbial communities of Khoormog, Chigee, and Airag, the TFDP from Xilin Gol in China, were investigated and compared. The physicochemical analysis revealed a higher content of total solid content, protein, and fat in Khoormog (12.5 ± 1.6%; 4.6 ± 0.7%; 4.4 ± 1.3%) compared to Chigee (7.8 ± 1.3%; 2.1 ± 0.2%; 0.8 ± 0.2%) and Airag (8.9 ± 0.7%; 3.7 ± 0.4%; 1.4 ± 0.5%). All three types of TFDP shared 41.2% of bacterial and 25.4% of fungal OTUs, and 95.34% of bacterial and 95.52% of fungal sequence reads. The bacterial and fungal community consisted of four phyla and five genera, and three phyla and seven genera, respectively. Lastly, *Lactobacillus* predominated in Khoormog, Chigee, and Airag at the genus level, while the dominant fungal genera varied among the samples. In conclusion, the microbial community structures of TFDP from camel, mare, and cow were not significantly different in a definite area (Xilingol region), and Khoormog, Chigee, and Airag bred the common “core microbiota”.

## Graphical abstract

This study is the first to investigate the nutritional properties and microbiota of Khoormog from camel's milk using high throughput sequencing. This study provides the comparative examination of the physicochemical composition and microbial community of Khoormog, Chigee, and Airag.

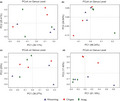

## 1 INTRODUCTION

In Inner Mongolia, the traditionally fermented dairy products (TFDP) from cow, mare, and camel play an important role in the lives of nomadic Mongolians. There are three kinds of spontaneously fermented dairy products made in Xilingol region, Inner Mongolia, China. The nomads from this region produce Khoormog, a spontaneously fermented dairy product with a sour and alcoholic taste from camel's milk, Chigee from mare's milk, and Airag from cow's milk. Raw camel, mare and cow milk was naturally cooled and filtered by multilayer gauze, and pretreatment raw milk was mixed with remaining TFDP or homemade starter culture to spontaneously ferment at ambient temperature (20–25°C). TFDP was produced after spontaneous fermentation for 1–2 days, and stirred up and down with a wooden stick to increase oxygen and ensure homogeneity. An ancient aseptic technique of natural fermentation was used to extend storage time of the milk and to produce TFDP with enriched nutritional properties and probiotics. The TFDP have a longstanding traditional consumption in many countries across the world due to their exceptional organoleptic properties and numerous health benefits. Several beneficial properties of the probiotics have been reported, such as anticancer, antihypertensive, antidiabetic, and antioxidant activities for fermented camel's milk (Ayyash et al., 2018), and angiotensin I‐converting enzyme (ACE) inhibitory activity for the traditionally fermented mare's milk (Chen et al., 2010). Moreover, the cholesterol‐lowering activity of *Lactobacillus helveticus* from fermented cow's milk and *L. fermentum* from fermented mare's milk was previously documented (Damodharan et al., 2016; Pan et al., 2011). More interestingly, certain aspects of research indicate that the physicochemical attributes and microbiota determine the versatile functions of these TFDP.

The physicochemical composition and microbial community were investigated in TFDP from mare and cow (Guo et al., 2019; Sun et al., 2014). However, there has been none of the researches on both nutritional properties and microbiota of Khoormog from camel's milk. Additionally, none of the studies reported the comparative examination of the physicochemical composition and microbial community of Khoormog, Chigee, and Airag. In this study, Khoormog, Chigee, and Airag from Xilingol region were gathered to investigate their physicochemical composition and microbial community.

## 2 MATERIALS AND METHODS

### 2.1 Sampling of TFDP

**FIGURE 1:**
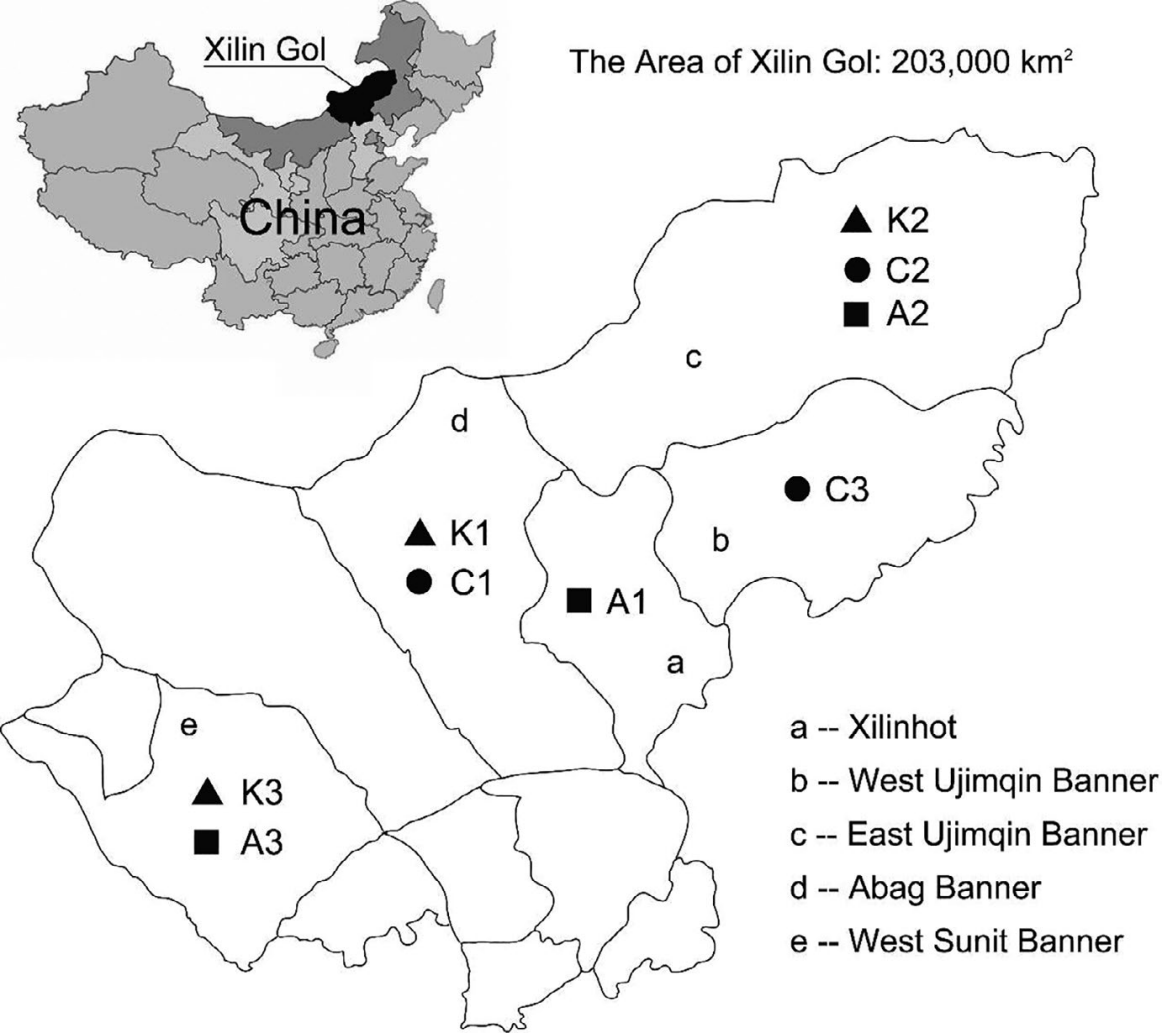
Sampling locations of TFDP from Xilin Gol in China. Khoormog (K1, K2, and K3) samples were collected from Abag Banner, East Ujimqin Banner, and West Sunit Banner. Chigee (C1, C2, and C3) samples were obtained from Abag Banner, East Ujimqin Banner, and West Ujimqin Banner. Airag (A1, A2, and A3) samples were acquired from Xilinhot, East Ujimqin Banner, and West Sunit Banner

The TFDP were collected from Xilin Gol in China. A total number of 9 samples of Khoormog (K1, K2, and K3), Chigee (C1, C2, and C3), and Airag (A1, A2, and A3) were obtained from nomads resided in East Ujimqin Banner, West Ujimqin Banner, Xilinhot, Abag Banner, and West Sunit Banner, respectively (Figure 1). These spontaneously fermented dairy products were immediately stored at 4℃ and analyzed to investigate the physicochemical composition and microbial community.

### 2.2 Physicochemical analysis

The total solid content (China National Food Safety Standard, 2016a), protein (China National Food Safety Standard, 2016b), fat (China National Food Safety Standard, 2016c), and alcohol content (China National Food Safety Standard, 2016d) was determined according to Chinese national food safety standards. The Kjeldahl method was used to measure protein content, and the Soxhlet extraction method for fat content; the lactose content was quantified using the titration method with the Feline's solution (Sinopharm) and methylene blue (Sinopharm) (China National Food Safety Standard, 2010). Acidity was determined by the titration method using Sodium hydroxide (0.1 mol/L) with a phenolphthalein indicator (Sinopharm) (China National Food Safety Standard, 2016e).

### 2.3 DNA Extraction, PCR amplification, sequencing, bioinformatics, and statistical analysis

Microbial genomic DNA was extracted from TFDP using the E.Z.N.A. soil DNA Kit (Omega Bio‐tek) according to manufacturer's instructions. DNA integrity was verified on 2% agarose gel, and DNA concentration and purity were determined with NanoDrop 2000 (Thermo Fisher Scientific). The hypervariable region V3‐V4 of the bacterial 16S rRNA gene was amplified with primer pairs 338F (5′‐ACTCCTACGGGAGGCAGCAG‐3′) and 806R (5′‐GGACTACHVGGGTWTCTAAT‐3′) by an ABI GeneAmp 9,700 PCR thermocycler (Applied Biosystems). The internal transcribed spacer (ITS) of the rRNA gene was amplified with the primer pairs ITS3F (5′‐GCATCGATGAAGAACGCAGC‐3′) and ITS4R (5′‐TCCTCCGCTTATTGATATGC‐3′). The PCR was performed as follows: initial denaturation at 95℃ for 3 min, followed by 37 cycles of denaturing at 95℃ for 30 s, annealing at 55℃ for 30 s, then extension at 72℃ for 45 s, final extension at 72℃ for 10 min, and finished at 4℃. The PCR mixtures contained 5 × 4 μl of TransStart FastPfu buffer (Transgen), 2 μl of 2.5 mM dNTPs (Transgen), 0.8 μl of forward primer (5 μM), 0.8 μl of reverse primer (5 μM), 0.4 μl of TransStart FastPfu DNA Polymerase (Transgen), 10 ng of template DNA, and finally ddH_2_O filled up to 20 μl. The PCR product was extracted from 2% agarose gel, purified using the AxyPrep DNA Gel Extraction Kit (Axygen Biosciences), and quantified using Quantus™ Fluorometer (Promega).

Purified amplicons were pooled in equimolar and paired‐end sequenced (2 × 300) on an Illumina MiSeq platform (Illumina). The raw sequencing reads were demultiplexed, quality‐filtered, and merged by FLASH. Operational taxonomic units (OTUs) with 97% similarity were clustered using UPARSE (version 7.1, http://drive5.com/uparse/), and chimeric sequences were identified and removed. The taxonomy of each OTU representative sequence was analyzed using RDP Classifier (http://rdp.cme.msu.edu/) against the 16S rRNA database (Silva) and ITS database (Unite) using the confidence threshold of 0.7. The microbial metabolic pathways analysis from the fermented dairy products was predicted based on the 16S rRNA and ITS data using phylogenetic investigation of communities by reconstruction of unobserved states 2 (PICRUSt2). The relationship between microbial genera and physicochemical indexes was assessed by Pearson correlation heatmap and considered statistically significant at *p* < .05.

## 3 RESULTS AND DISCUSSION

### 3.1 Physicochemical analysis of Khoormog, Chigee, and Airag

The physicochemical analysis was applied to evaluate the nutritional properties of TFDP from three animals in Xilin Gol, China. The values of total solid content (12.5 ± 1.6%), protein (4.6 ± 0.7%), and fat (4.4 ± 1.3%) in Khoormog were higher than in both Chigee (7.8 ± 1.3%; 2.1 ± 0.2%; 0.8 ± 0.2%) and Airag (8.9 ± 0.7%; 3.7 ± 0.4%; 1.4 ± 0.5%) (Table 1). Khoormog, Chigee, and Airag contained lactose of 2.1 ± 1.2%, 4.2 ± 1.6%, and 2.4 ± 0.4%, respectively. The lactose contents of these TFDP were lower compared to those reported in fresh camel, mare, and cow milk (Tsakalidou & Papadimitriou, 2016). During the process of spontaneous dairy fermentation, the lactic acid bacteria (LAB) use lactose as a source of carbon and produce lactic acid, which increases the acidity of fermented products, while yeasts enhance the alcohol content. The acidity of Khoormog, Chigee, and Airag were 244.1 ± 62.5°T, 168 ± 50.6°T, and 238.5 ± 89.1°T, and the content of alcohol were 0.2 ± 0.2% for Khoormog, 0.5 ± 0.7% for Chigee, and 0.1 ± 0.1% for Airag (Table 1).

**TABLE 1:** Physicochemical composition of Khoormog, Chigee, and Airag from Xilin Gol in China

Sample	Total solid content (g/100 g)	Protein (g/100 g)	Fat (g/100 g)	Lactose (g/100 g)	Acidity^a^ (°T)	Alcohol (g/100 g)
K1	13.65 ± 0.03	3.65 ± 0.03	5.94 ± 0.03	2.83 ± 0.01	187.3 ± 0.6	0
K2	13.43 ± 0.31	5.09 ± 0.01	3.07 ± 0.003	0.45 ± 0.003	325.3 ± 0.6	0.37 ± 0.23
K3	10.35 ± 0.03	5.12 ± 0.04	4.25 ± 0.01	2.97 ± 0.01	219.6 ± 0.7	0.14 ± 0.05
C1	6.3 ± 0.1	1.86 ± 0.01	0.87 ± 0.01	4.43 ± 0.02	224.7 ± 0.1	0.04 ± 0.04
C2	7.86 ± 0.13	2.28 ± 0.01	0.56 ± 0.01	5.9 ± 0.04	107.9 ± 0.1	0.09 ± 0.04
C3	9.36 ± 0.01	2.05 ± 0.08	0.94 ± 0.02	2.15 ± 0.01	171.4 ± 0.1	1.4 ± 0.02
A1	8.93 ± 0.16	4.13 ± 0.07	0.73 ± 0.02	2.26 ± 0.01	282.3 ± 1	0.16 ± 0.07
A2	9.35 ± 0.05	3.81 ± 0.03	1.45 ± 0.06	2.07 ± 0.01	312.1 ± 0.7	0
A3	8.45 ± 1.09	3.12 ± 0.03	1.9 ± 0.01	2.85 ± 0.01	121 ± 0.6	0.23 ± 0.13

^a^ Degrees Theurer.

A relative abundance of macromolecular compounds in Khoormog may contribute to higher antioxidant activity, angiotensin‐converting enzyme inhibition, and anticancer activity in comparison to compounds from fermented bovine milk (Ayyash et al., 2018). Thus, the derivatives and metabolites of protein and fat in TFDP are considered indicators of executive functions, such as antihypertension, antioxidant activity, and anticancer activity. The samples of spontaneously fermented products, Khoormog, Chigee, and Airag were obtained from Mongolian herdsmen in yurts across the entire grassland from five administrative divisions of Xilin Gol, China. According to the obtained values of *SD* (Table 1), we observed large differences in acidity, lactose and alcohol content, between Khoormog, Chigee, and Airag. Presumably, the diversity of starter culture, operational habit of individual, and humiture and hygiene of yurt may determine the consumption of lactose by lactic acid bacteria and yeasts, and thus, the quality features of Khoormog, Chigee, and Airag, such as acidity and alcohol content. Therefore, these huge differences reflected the natural quality of homemade fermented milk in a wide range of variations.

### 3.2 Bacterial and fungal sequence reads analysis

The 16S rRNA gene was amplified from three Khoormog, three Chigee, and three Airag samples. A total of 566,227 of high‐quality bacterial sequence reads were generated from the nine traditionally fermented samples, with an average of 62,914 (*SD* = 5,816) (Table 2). Afterward, the ITS gene was amplified from these samples, and a total of 994,354 of high‐quality fungal sequence reads were generated, with an average of 110,484 (*SD* = 16,831) (Table 2). The high‐quality sequence reads were clustered into 68 OTUs for bacteria and 59 OTUs for fungi, with an average of 29.8 ± 6.8 and 17.6 ± 6.4 per sample, respectively (Table 2).

**TABLE 2:** Diversity indexes of 16S rRNA and ITS sequencing of Khoormog, Chigee, and Airag from Xilin Gol in China

Target	Sample	No. of reads	No. of OTUs^a^	Chao1 index	Shannon index	Simpson index	Good's coverage
Bacterial analysis	K1	71,740	35	35	1.80	0.20	0.99995
	K2	55,567	20	22	1.13	0.34	0.99992
	K3	52,767	33	35	1.11	0.43	0.99992
	C1	63,804	22	24	0.54	0.77	0.99995
	C2	61,477	29	31	1.14	0.52	0.99991
	C3	67,144	28	31	1.49	0.34	0.99994
	A1	62,489	25	26	0.65	0.73	0.99995
	A2	65,960	35	37	1.06	0.50	0.99992
	A3	65,279	41	49	0.94	0.60	0.99990
Fungal analysis	K1	121,898	16	22	0.49	0.73	0.99997
	K2	119,768	10	13	0.62	0.69	0.99998
	K3	92,949	17	19	0.72	0.55	0.99996
	C1	123,809	32	43	0.81	0.62	0.99992
	C2	82,914	18	19	0.37	0.84	0.99998
	C3	89,470	15	16	0.46	0.74	0.99997
	A1	120,416	20	22	0.39	0.79	0.99997
	A2	124,633	11	14	0.02	1.00	0.99998
	A3	118,497	19	19	0.97	0.52	0.99998

^a^ Operational taxonomic units.

**FIGURE 2:**
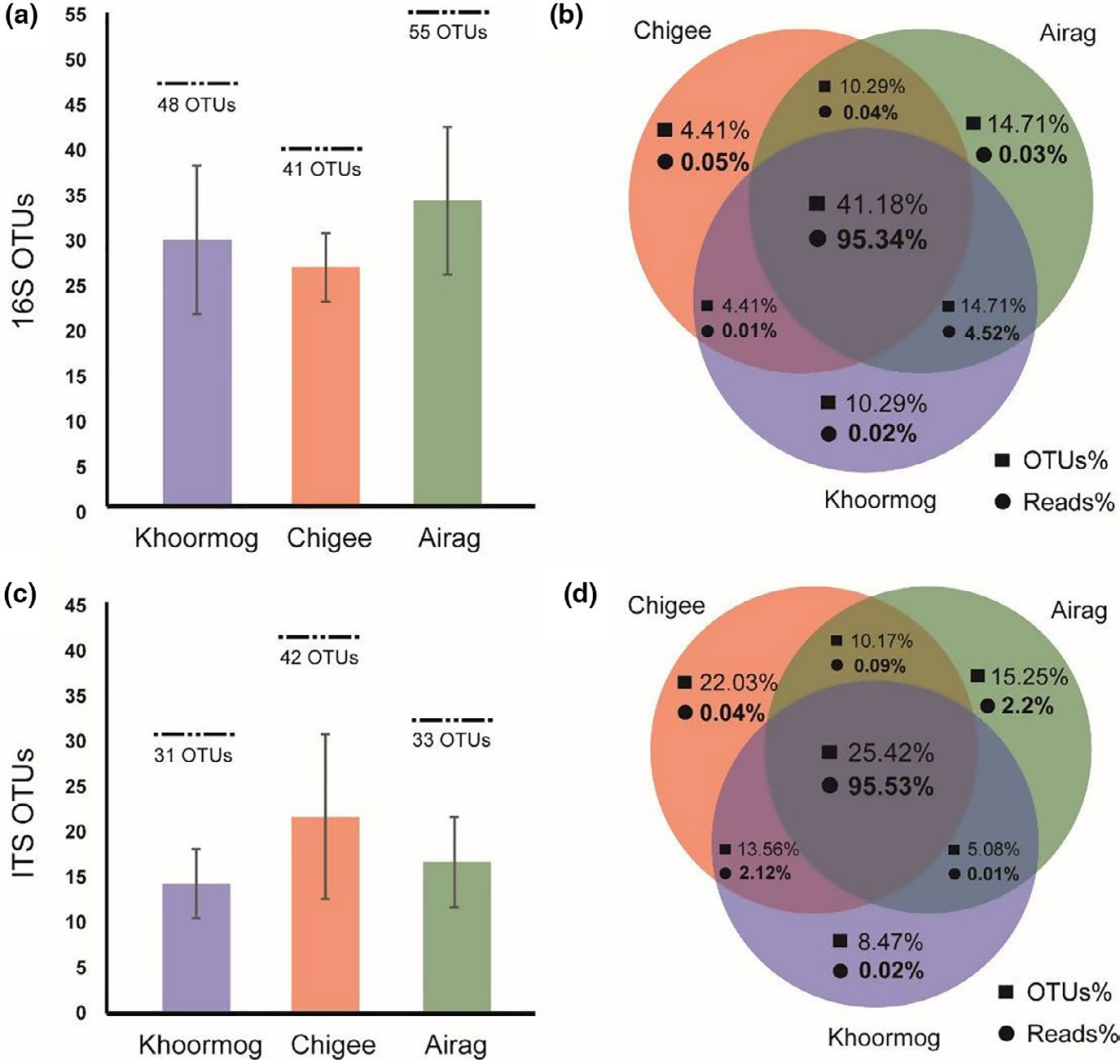
Analyses of abundances of OTUs and sequence read from the bacterial and fungal community. The average and a total number of OTUs (a and c), the percentages of OTUs and sequence reads (b and d) for 16S rRNA (a and b), and ITS (c and d) sequencing analyses in Khoormog, Chigee, and Airag are presented. OTUs = operational taxonomic units. The color version is available online

Regarding the differences between the samples of three different animals, the number of bacterial OTUs was 29.3 ± 8.1 for Khoormog, 26.3 ± 3.8 for Chigee, and 33.7 ± 8.1 for Airag. A total of 48, 41, and 55 OTUs identified in these dairy products is shown in Figure 2a. In addition, the number of fungal OTUs was 14.3 ± 3.8 for camel samples, 21.7 ± 9.1 for mare samples, and 16.7 ± 4.9 for cow samples, and a total of 31, 42, and 33 OTUs identified in these samples are presented in Figure 2c. We found that 70.6% of bacterial and 54.2% of fungal OTUs were shared by at least two types of these dairy products, whereas 41.2% of bacterial and 25.4% of fungal OTUs were shared by all three types (Figure 2b,d). Interestingly, the two of these dairy products shared 99.91% of bacterial and 97.74% of fungal sequence reads, while all three types shared 95.34% and 95.52% of bacterial and fungal sequence reads, respectively (Figure 2b,d). The three types of analyzed dairy products from camel, mare, and cow shared microbial OTUs to a certain extent, while the OTUs sequence reads predominantly originated from bacterial and fungal reads. Regarding the similarity of geographical origin (Xilingol region in Inner Mongolia) and artisanal technology of these traditional dairy products, we hypothesized that these naturally fermented dairy products within a certain geographic area could share the common functional “core microbiota” in the process of spontaneous fermentation. This also implies that the distinct components in different animal milk may contribute to the enrichment of specific microorganisms (OTUs).

The analysis of alpha diversity for the bacterial and fungal reads was performed on Khoormog, Chigee, and Airag. As shown in Figure S1, the rarefaction curves maintained an exponential increase; however, the Shannon diversity curves reached the saturation phase. Although the analysis of alpha diversity revealed that the majority of bacterial and fungal OTUs for Khoormog, Chigee, and Airag samples have already been reported, several unidentified bacterial and fungal OTUs could possibly be determined by increasing the depth of sequencing. Nevertheless, the majority of bacterial and fungal OTUs have already been identified in the three types of dairy products using the Good's coverage (values >99%).

The microbial richness between bacterial and fungal communities of TFDP was compared using the number of OTUs and Chao index. The bacterial number of OTUs (29.8 ± 6.8) and Chao index (32.1 ± 8.2) were significantly higher than in fungal samples (17.6 ± 6.4; 20.7 ± 9.0) (*p* < .01; *p* < .05). On the other hand, the microbial diversity was estimated using Shannon and Simpson indexes. The bacterial Shannon index (1.10 ± 0.39) was significantly higher than fungal (0.54 ± 0.28) (*p* < .05), while the bacterial Simpson index (0.49 ± 0.19) was significantly lower than fungal (0.72 ± 0.15) (*p* < .05). Therefore, evaluations of microbial richness showed greater variety and diversity of bacteria compared to fungal communities in TFDP from Xilingol region in Inner Mongolia.

The analysis of alpha diversity of TFDP (Khoormog, Chigee, and Airag) originated from camel, mare, and cow, collected from Xilingol region in Inner Mongolia, China was also performed to compare the microbial variety and diversity. The results of statistical analysis demonstrated that there was no difference between these three types of samples (*p* > .05). Thus, it may be presumed that milk's origin could not be used to determine bacterial and fungal variety and diversity of naturally fermented dairy products.

### 3.3 Bacterial community structure analysis

The relative abundance analysis of bacterial taxa, based on the 16S rRNA sequences, showed that in these nine samples four phyla could be identified: *Firmicutes* (relative abundance: 82.74%), *Proteobacteria* (17.24%), *Actinobacteria* (0.01%), and *Bacteroidetes* (0.01%). Furthermore, *Firmicutes* dominated in all samples, representing 77.15%, 77.05%, 90.39%, 91.42%, 75.91%, 56.45%, 92.58%, 92.11%, and 92.25% of the sequence reads from Khoormog, Chigee, and Airag from Xilingol region, respectively. In the previous researches of traditionally fermented products (vrum, koumiss, and tarag), the identical four phyla had been identified as major phyla, along with the predominance of *Firmicutes* (Guo et al., 2019; Sun et al., 2014; Yamei et al., 2019).

**FIGURE 3:**
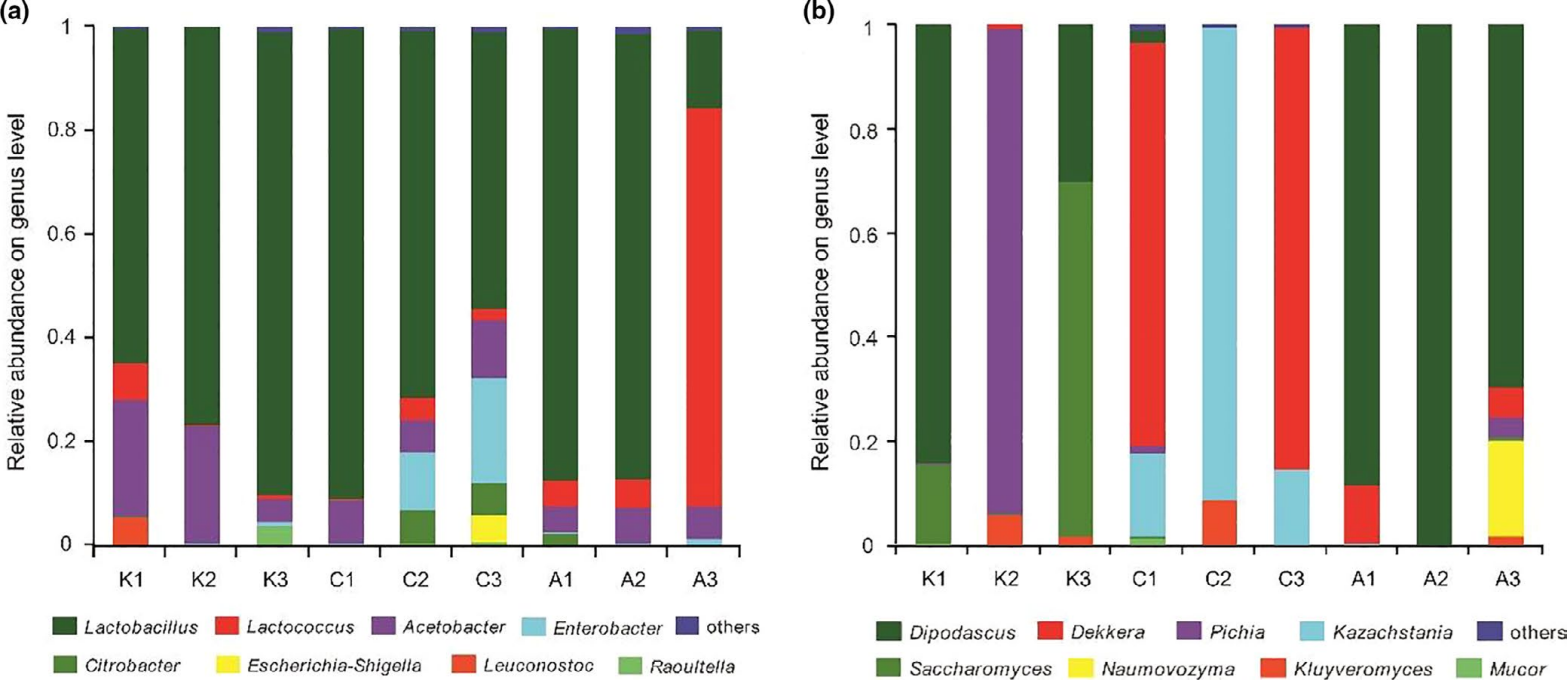
The relative abundance of bacterial (a) and fungal (b) sequence in the nine traditionally fermented dairy samples of Khoormog, Chigee, and Airag. The color version is available online

As shown in Figure 3a, five bacterial genera were identified in these nine samples, with an average relative abundance >1%. The relative abundances of genera were 11.78% for *Lactococcus*, 10.27% for *Acetobacter*, 3.94% for *Enterobacter*, and 1.75% for *Citrobacter*; *Lactobacillus* (70.55%) predominated at the level of genus. Specifically, *Lactobacillus* dominated in K1, K2, K3, C1, C2, C3, A1, and A2 corresponding to 64.55%, 76.84%, 89.51%, 90.83%, 71.06%, 53.81%, 87.19%, and 86.34%, respectively. *Lactococcus* was the predominant bacterial genus in A3 with an average relative abundance of 77.04%. Regarding the differences of the origin between the samples, the bacterial community of Khoormog (camel) consisted of *Lactobacillus* (76.97%), *Acetobacter* (16.53%), *Lactococcus* (2.75%), *Leuconostoc* (1.73%), and *Raoultella* (1.21%). Chigee (mare) contained *Lactobacillus* (71.9%), *Enterobacter* (10.7%), *Acetobacter* (8.36%), *Citrobacter* (4.25%), *Lactococcus* (2.28%), and *Escherichia‐Shigella* (1.75%). Airag (cow) consisted of *Lactobacillus* (62.78%), *Lactococcus* (29.12%), and *Acetobacter* (6.08%). Previous studies reported that *Lactobacillus* and *Lactococcus* represent the major genera in TFDP, while *Acetobacter*, *Enterobacter*, and *Leuconostoc* also comprise the bacterial community in dairy products, such as fermented vrum (Yamei et al., 2019), koumiss (Guo et al., 2019), tarag (Sun et al., 2014), hurood cheese, and jueke (Gao et al., 2017). In addition, a very small amount of contamination with *Citrobacter*, *Raoultella*, and *Escherichia‐Shigella* was detected in several samples. This instance of bacterial contamination may result due to nonpasteurization of raw milk, insanitation of tools, or artisanal fermentation.

**FIGURE 4:**
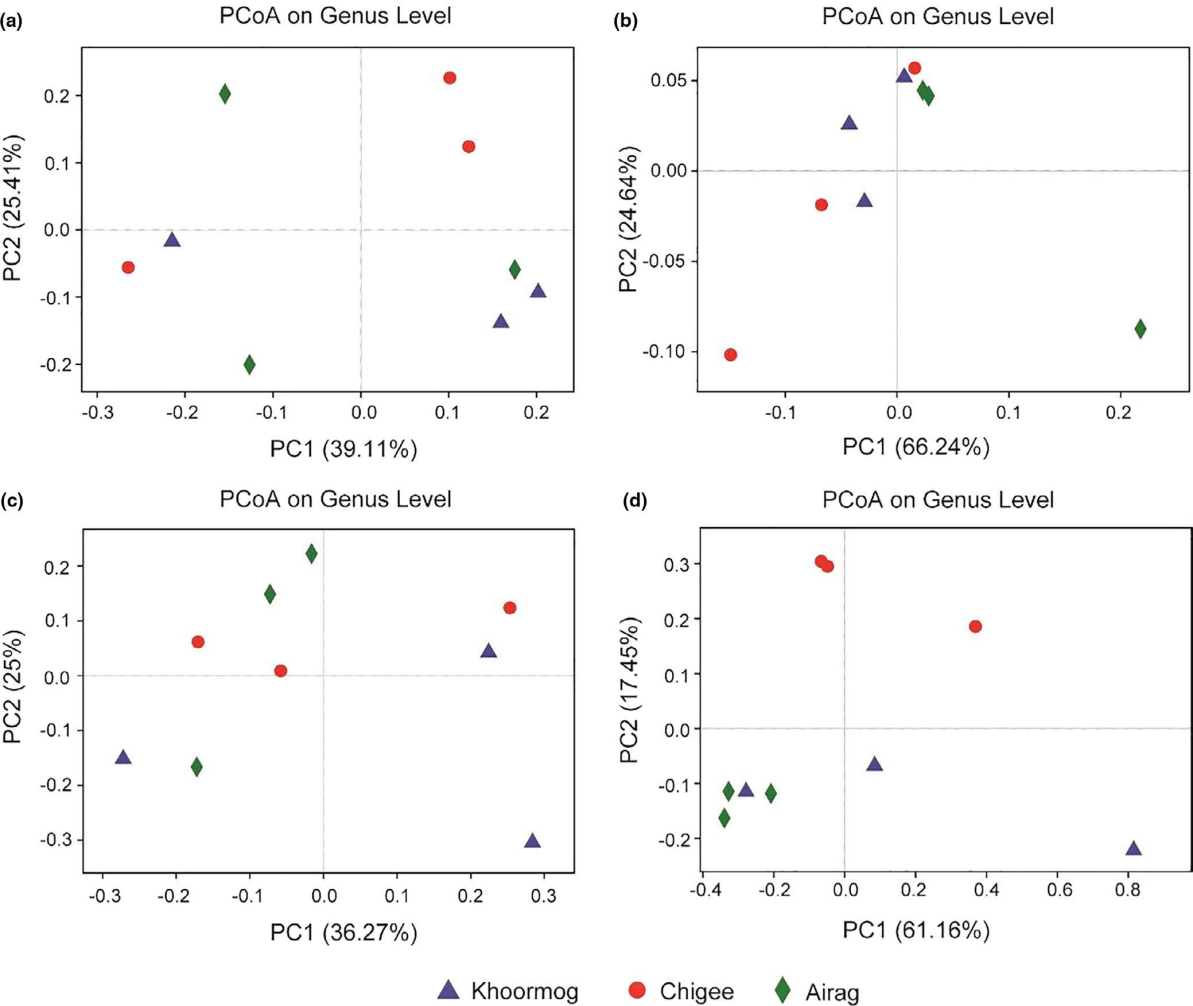
Unweighted (a and c) and weighted (b and d) UniFrac principal coordinate analyses of the bacterial (a and b) and fungal (c and D) diversity in Khoormog, Chigee, and Airag from Xilin Gol in China. The blue (trilateral), red (roundness), and green (tetragonal) symbols represent Khoormog, Chigee, and Airag, respectively. The color version is available online

**FIGURE 5:**
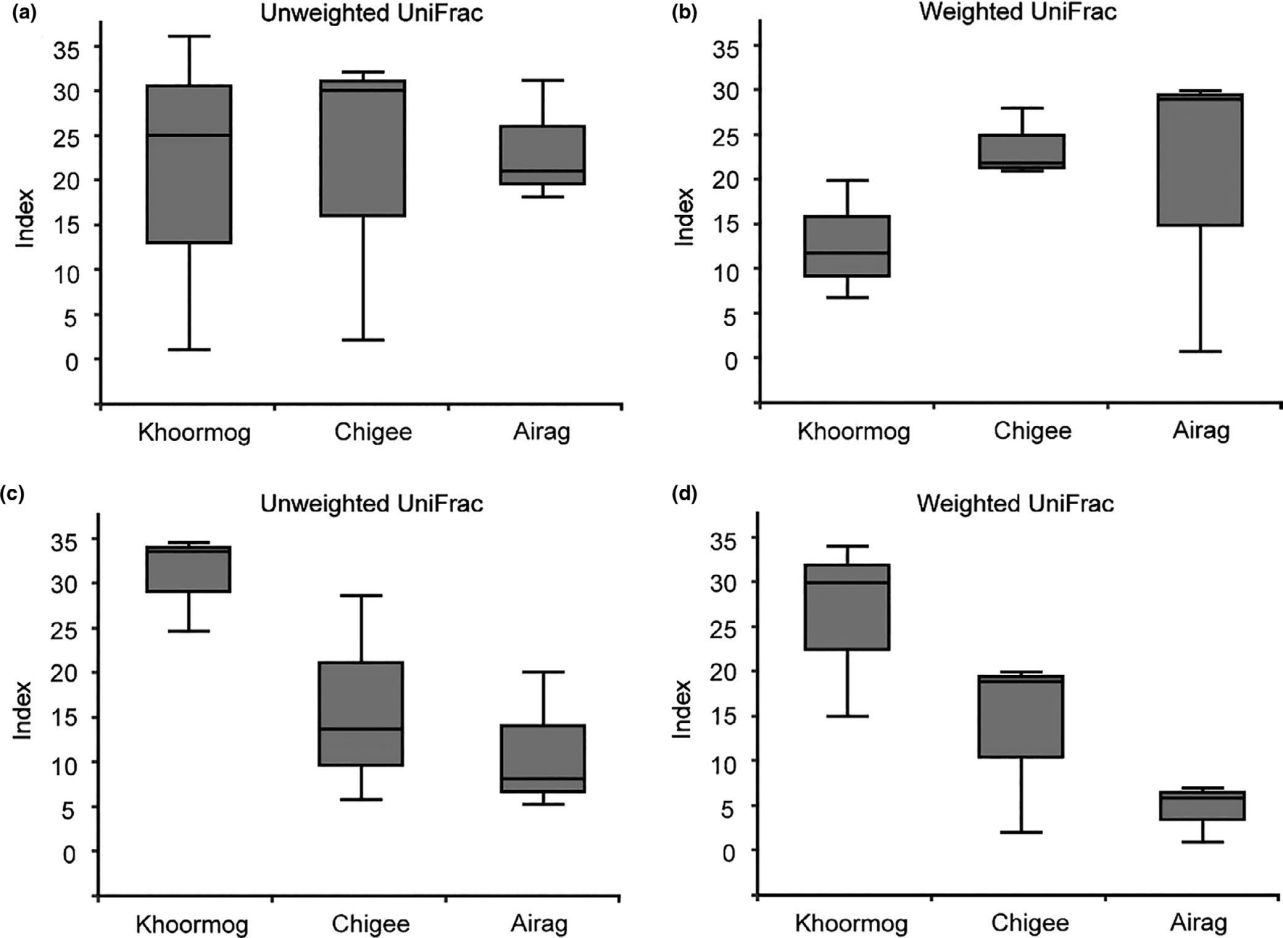
The Anosim analysis of unweighted (a and c) and weighted (b and d) UniFrac principal coordinate analyses of the bacterial (a and b) and fungal (c and d) diversity in Khoormog, Chigee, and Airag from Xilin Gol in China

The bacterial community structures of TFDP from camel, mare, and cow were analyzed using the unweighted and weighted UniFrac PCoA. The results showed that nine samples from three animals were largely separated in the both unweighted (corresponding to 39.11% and 25.41% of the total variance by the two principal components, respectively) and weighted (corresponding to 66.24% and 24.64% of the total variance by the two principal components, respectively) analysis (Figure 4a,b). In addition, the Anosim analysis, based on the unweighted and weighted UniFrac, was also utilized to evaluate the differences in the bacterial community of fermented dairy products from three animals. As shown in Figure 5a,b, the results demonstrated that there was no difference in the bacterial community between the samples from three animals (R = −0.029; *p* = .561). Thus, the existence of “core microbiota” in these three types (camel, mare, and cow) of TFDP was supported with the results obtained from UniFrac PCoA and Anosim analyses.

### 3.4 Fungal community structure analysis

The analysis of fungal taxa relative abundances, based on the ITS sequences, showed that in these nine samples three phyla could be identified: *Ascomycota* (99.73%), *Zygomycota* (17.24%), and *Basidiomycota* (0.01%). These results are consistent with the previous investigations where identical three fungal phyla were identified as well as the predominance of *Firmicutes* in traditionally fermented vrum and koumiss from Xilingol region (Guo et al., 2019; Sun et al., 2014; Yamei et al., 2019).

As shown in Figure 3b, in these nine samples, seven fungal genera have been identified, with an average relative abundance >1%. The relative abundances of genera were *Dipodascus* (45.09%), *Dekkera* (19.32%), *Pichia* (11.91%), *Kazachstania* (10.97%), *Saccharomyces* (8.37%), *Naumovozyma* (2.19%), and *Kluyveromyces* (1.76%). In addition, *Dipodascus* dominated in K1, A1, A2, and A3, corresponding to 84.21%, 88.38%, 99.94%, and 69.51%, respectively. *Dekkera* was the predominant fungal genus in C1 and C3 with the relative abundances of 77.40% and 85.17%. *Pichia*, *Kazachstania*, and *Saccharomyces* dominated in K2 (93.46%), C2 (91.18%), and K3 (68.22%), respectively. Regarding the origin of the samples from three different animals, the fungal community of Khoormog consisted of *Dipodascus* (38.05%), *Pichia* (31.24%), *Saccharomyces* (27.91%), and *Kluyveromyces* (2.44%). Chigee contained *Dekkera* (54.19%), *Kazachstania* (40.56%), and *Kluyveromyces* (2.84%). Airag consisted of *Dipodascus* (62.78%), *Naumovozyma* (6.17%), *Dekkera* (5.72%), and *Pichia* (1.28%). These genera are common components of the fungal TFDP community (Guo et al., 2019; Sun et al., 2014; Yamei et al., 2019; Gao et al., 2017). The results obtained from the relative abundances of genera in fungal communities showed that the dominant fungal genera varied among different samples.

The analysis of the fungal communities of TFDP (from camel, mare, and cow) was performed using the unweighted and weighted UniFrac PCoA. The obtained values of nine samples from three animals diverged largely in both unweighted (accounting for 36.27% and 25% of the total variance by the two principal components, respectively) and weighted (accounting for 61.16% and 17.45% of the total variance by the two principal components, respectively) analysis (Figure 4c,d). In addition, the unweighted and weighted UniFrac Anosim analysis of the sequence reads was used to evaluate the differences between the fungal communities of TFPD from camel, mare, and cow. The results showed that there was no difference between the three types of naturally fermented dairy products (R = 0.268; *p* = .062) (Figure 5c,d). Hence, these results support the previously stated assumption of the “core microbiota” in three types of TFDP from camel, mare, and cow.

### 3.5 Metabolic pathways and correlation analyses

**FIGURE 6:**
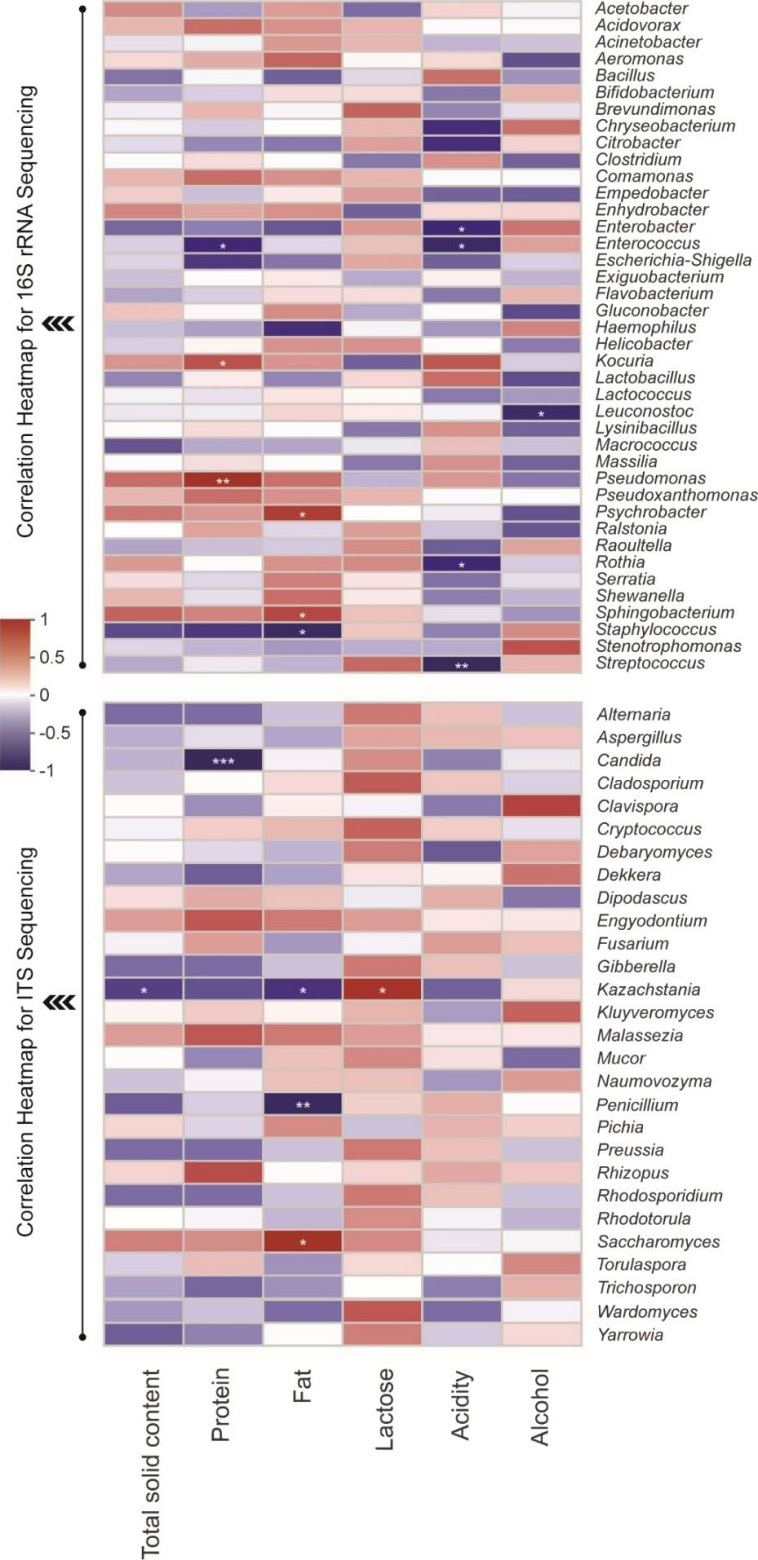
Spearman's correlation heatmap between different bacterial and fungal genera and physicochemical indexes

The microbial metabolic pathways of the traditional fermented dairy products were predicted to compare the different microbial pathways among the nine samples from camel, mare, and cow. The metabolic pathways were adenosine deoxyribonucleotides de novo biosynthesis II, guanosine deoxyribonucleotides de novo biosynthesis II, gondoate biosynthesis (anaerobic), pyruvate fermentation to acetate and lactate II, cis‐vaccenate biosynthesis, and so on for 16S rRNA data prediction (Table S1) and aerobic respiration I (cytochrome c), aerobic respiration II (cytochrome c) (yeast), glyoxylate cycle, TCA cycle II (plants and fungi), adenosine ribonucleotides de novo biosynthesis, and so on for ITS data prediction (Table S2), and these above predicted metabolic pathways were enriched in each type of samples. To explore the potentially functional microbial profile in traditional fermented dairy products, we calculated all pairwise Spearman's correlations between different bacterial and fungal genera and physicochemical indexes, and constructed a correlation heatmap. We found some bacterial and fungal genera to do with total solid content, protein, fat, lactose, acidity, and alcohol (Figure 6). Nevertheless, only 3 samples in each group are not enough to elucidate the correlations between microbial genera and physicochemical indexes. In future, we will try our best to collect enough samples to investigate the differences of the physicochemical composition and microbial communities between Khoormog, Chigee, and Airag from Xilin Gol in the same family (yurt) at the same time in order to study the influence of the types of milk on microbial community, and we use homemade starters to inoculate pasteurized raw milk from camel, mare, and cow to study the influence of the types of milk on microbial community.

## 4 CONCLUSIONS

Four phyla (*Firmicutes*, *Proteobacteria*, *Actinobacteria*, and *Bacteroidetes*) and five genera (*Lactobacillus*, *Lactococcus*, *Acetobacter*, *Enterobacter*, and *Citrobacter*) constituted the bacterial community of Khoormog, Chigee, and Airag, where *Lactobacillus* was a dominant genus. In addition, the fungal community of Khoormog, Chigee, and Airag consisted of three phyla (*Ascomycota*, *Zygomycota*, and *Basidiomycota*) and seven genera (*Dipodascus*, *Dekkera*, *Pichia*, *Kazachstania*, *Saccharomyces*, *Naumovozyma*, and *Kluyveromyces*), and the dominant genera varied among samples. Although Khoormog, Chigee, and Airag shared 41.2% of bacterial and 25.4% of fungal OTUs, the microbial community structures of TFDP from camel, mare, and cow were not significantly different. The similarity between microbial communities of Khoormog, Chigee, and Airag may result from a large percentage of shared bacterial (95.34%) and fungal (95.52%) sequence reads occupied by bacterial and fungal OTUs. The results demonstrated that these naturally fermented dairy products within a certain geographic area could share the common functional “core microbiota” in the fermentation process, and the distinct components in different animal milk may contribute to the enrichment of specific microorganisms.

## CONFLICT OF INTEREST

All authors declare no conflict of interest.

## Supporting information

App S1

## Data Availability

The data that support the findings of this study are available from the corresponding author upon reasonable request.

## References

[fsn32131-bib-0001] Ayyash, M. , Al‐Dhaheri, A. S. , Al Mahadin, S. , Kizhakkayil, J. , & Abushelaibi, A. (2018). In vitro investigation of anticancer, antihypertensive, antidiabetic, and antioxidant activities of camel milk fermented with camel milk probiotic: A comparative study with fermented bovine milk. Journal of Dairy Science, 101, 900–911. 10.3168/jds.2017-13400 29224862

[fsn32131-bib-0002] Chen, Y. , Wang, Z. , Chen, X. , Liu, Y. , Zhang, H. , & Sun, T. (2010). Identification of angiotensin I‐converting enzyme inhibitory peptides from koumiss, a traditional fermented mare's milk. Journal of Dairy Science, 93, 884–892. 10.3168/jds.2009-2672 20172208

[fsn32131-bib-0003] China National Food Safety Standard (2010). GB 5413.5‐2010. Determination of lactose and sucrose in foods for infants and young children. Ministry of Health of the People's Republic of China, China: National food safety standard.

[fsn32131-bib-0004] China National Food Safety Standard (2016a). GB 5009.3‐2016. Determination of moisture in foods. Ministry of Health of the People's Republic of China, China: National food safety standard.

[fsn32131-bib-0005] China National Food Safety Standard (2016b). GB 5009.5‐2016. Determination of protein in foods. Ministry of Health of the People's Republic of China, China: National food safety standard.

[fsn32131-bib-0006] China National Food Safety Standard (2016c). GB 5009.6‐2016. Determination of fat in foods. Ministry of Health of the People's Republic of China, China: National food safety standard.

[fsn32131-bib-0007] China National Food Safety Standard (2016d). GB 5009.225‐2016. Determination of alcohol in liquors. Ministry of Health of the People's Republic of China, China: National food safety standard.

[fsn32131-bib-0008] China National Food Safety Standard (2016e). GB 5009.239‐2016. Determination of acidity in foods. Ministry of Health of the People's Republic of China, China: National food safety standard.

[fsn32131-bib-0009] Damodharan, K. , Palaniyandi, S. A. , Yang, S. H. , & Suh, J. W. (2016). Functional probiotic characterization and in vivo cholesterol‐lowering activity of *Lactobacillus helveticus* isolated from fermented cow milk. Journal of Microbiology and Biotechnology, 26, 1675–1686. 10.4014/jmb.1603.03005 27435541

[fsn32131-bib-0010] Gao, M. L. , Hou, H. M. , Teng, X. X. , Zhu, Y. L. , Hao, H. S. , & Zhang, G. L. (2017). Microbial diversity in raw milk and traditional fermented dairy products (Hurood cheese and Jueke) from Inner Mongolia, China. Genet Mol Res, 16(1). 10.4238/gmr16019451 28290619

[fsn32131-bib-0011] Guo, L. , Ya, M. , Guo, Y. S. , Xu, W. L. , Li, C. D. , Sun, J. P. , Zhu, J. J. , & Qian, J. P. (2019). Study of bacterial and fungal community structures in traditional koumiss from Inner Mongolia. Journal of Dairy Science, 102, 1972–1984. 10.3168/jds.2018-15155 30639001

[fsn32131-bib-0012] Pan, D. D. , Zeng, X. Q. , & Yan, Y. T. (2011). Characterization of *Lactobacillus fermentum* SM‐7 isolated from koumiss, a potential probiotic bacterium with cholesterol‐lowering effects. Journal of the Science of Food and Agriculture, 91, 512–518. 10.1002/jsfa.4214 21218486

[fsn32131-bib-0013] Sun, Z. , Liu, W. , Bao, Q. , Zhang, J. , Hou, Q. , Kwok, L. , Sun, T. , & Zhang, H. (2014). Investigation of bacterial and fungal diversity in tarag using high‐throughput sequencing. Journal of Dairy Science, 97, 6085–6096. 10.3168/jds.2014-8360 25129502

[fsn32131-bib-0014] Tsakalidou, E. , & Papadimitriou, K. (2016). Non‐Bovine Milk and Milk Products. Elsevier.

[fsn32131-bib-0015] Yamei , Guo, Y. S. , Zhu, J. J. , Xiao, F. , Hasiqimuge , Sun, J. P. , Qian, J. P. , Xu, W. L. , Li, C. D. , & Guo, L. (2019). Investigation of physicochemical composition and microbial communities in traditionally fermented vrum from Inner Mongolia. Journal of Dairy Science, 102, 8745–8755. 10.3168/jds.2019-16288 31400900

