# Supplementary material for: Comparative study of physicochemical composition and microbial community of Khoormog, Chigee, and Airag, traditionally fermented dairy products from Xilin Gol in China
Source: Food Sci Nutr. 2021 Jan 19;9(3):1564–73. doi: 10.1002/fsn3.2131 (PMC7958581; doi:10.1002/fsn3.2131)
Supplement: Supplementary file 1 — App S1 [file FSN3-9-1564-s001.docx]

Supplemental Table S1. Relative abundance (%) of metabolic pathway of bacterial genes in the traditionally fermented dairy products (TFDP) from camel, mare, and cow acquired from phylogenetic investigation of communities by reconstruction of unobserved states 2 (PICRUSt2).

| Metabolic pathway | K1 | K2 | K3 | C1 | C2 | C3 | A1 | A2 | A3 |
| --- | --- | --- | --- | --- | --- | --- | --- | --- | --- |
| Adenosine deoxyribonucleotides de novo biosynthesis II | 0.010 | 0.014 | 0.019 | 0.025 | 0.017 | 0.013 | 0.022 | 0.020 | 0.010 |
| Guanosine deoxyribonucleotides de novo biosynthesis II | 0.010 | 0.014 | 0.019 | 0.025 | 0.017 | 0.013 | 0.022 | 0.020 | 0.010 |
| Gondoate biosynthesis (anaerobic) | 0.014 | 0.014 | 0.016 | 0.018 | 0.013 | 0.011 | 0.017 | 0.016 | 0.016 |
| Pyruvate fermentation to acetate and lactate II | 0.012 | 0.014 | 0.017 | 0.020 | 0.014 | 0.011 | 0.018 | 0.017 | 0.012 |
| Cis-vaccenate biosynthesis | 0.012 | 0.014 | 0.016 | 0.019 | 0.014 | 0.012 | 0.017 | 0.017 | 0.012 |
| Superpathway of pyrimidine nucleobases salvage | 0.011 | 0.013 | 0.016 | 0.019 | 0.013 | 0.011 | 0.017 | 0.016 | 0.012 |
| Fatty acid elongation -- saturated | 0.012 | 0.013 | 0.015 | 0.018 | 0.013 | 0.011 | 0.016 | 0.015 | 0.013 |
| Superpathway of adenosine nucleotides de novo biosynthesis I | 0.010 | 0.012 | 0.015 | 0.017 | 0.012 | 0.010 | 0.015 | 0.015 | 0.010 |
| Superpathway of adenosine nucleotides de novo biosynthesis II | 0.010 | 0.012 | 0.014 | 0.017 | 0.012 | 0.010 | 0.015 | 0.014 | 0.010 |
| Acetylene degradation | 0.009 | 0.012 | 0.016 | 0.014 | 0.011 | 0.009 | 0.013 | 0.014 | 0.012 |
| Bifidobacterium shunt | 0.009 | 0.013 | 0.016 | 0.016 | 0.010 | 0.008 | 0.014 | 0.014 | 0.010 |
| CDP-diacylglycerol biosynthesis II | 0.010 | 0.012 | 0.014 | 0.016 | 0.011 | 0.010 | 0.014 | 0.013 | 0.010 |
| CDP-diacylglycerol biosynthesis I | 0.010 | 0.012 | 0.014 | 0.016 | 0.011 | 0.010 | 0.014 | 0.013 | 0.010 |
| 5-aminoimidazole ribonucleotide biosynthesis II | 0.010 | 0.012 | 0.014 | 0.016 | 0.011 | 0.009 | 0.014 | 0.014 | 0.009 |
| Superpathway of 5-aminoimidazole ribonucleotide biosynthesis | 0.010 | 0.012 | 0.014 | 0.016 | 0.011 | 0.009 | 0.014 | 0.014 | 0.009 |
| UMP biosynthesis | 0.010 | 0.012 | 0.013 | 0.015 | 0.011 | 0.009 | 0.013 | 0.013 | 0.010 |
| Glycolysis II (from fructose 6-phosphate) | 0.009 | 0.009 | 0.012 | 0.016 | 0.012 | 0.010 | 0.014 | 0.013 | 0.011 |
| Adenosine ribonucleotides de novo biosynthesis | 0.009 | 0.011 | 0.013 | 0.015 | 0.011 | 0.009 | 0.013 | 0.013 | 0.010 |
| 5-aminoimidazole ribonucleotide biosynthesis I | 0.009 | 0.011 | 0.013 | 0.015 | 0.010 | 0.009 | 0.013 | 0.013 | 0.009 |
| Peptidoglycan maturation (meso-diaminopimelate containing) | 0.009 | 0.011 | 0.014 | 0.013 | 0.010 | 0.009 | 0.012 | 0.013 | 0.011 |
| Guanosine ribonucleotides de novo biosynthesis | 0.009 | 0.011 | 0.013 | 0.015 | 0.010 | 0.009 | 0.013 | 0.013 | 0.009 |
| Homolactic fermentation | 0.008 | 0.008 | 0.012 | 0.016 | 0.011 | 0.009 | 0.014 | 0.013 | 0.011 |
| Superpathway of phospholipid biosynthesis I (bacteria) | 0.009 | 0.011 | 0.013 | 0.015 | 0.010 | 0.009 | 0.013 | 0.013 | 0.010 |
| Glycolysis III (from glucose) | 0.009 | 0.010 | 0.012 | 0.015 | 0.011 | 0.009 | 0.013 | 0.012 | 0.010 |
| Superpathway of L-threonine biosynthesis | 0.010 | 0.011 | 0.013 | 0.014 | 0.010 | 0.009 | 0.013 | 0.013 | 0.008 |
| L-lysine biosynthesis III | 0.010 | 0.011 | 0.013 | 0.014 | 0.010 | 0.009 | 0.013 | 0.012 | 0.008 |
| Sucrose degradation III (sucrose invertase) | 0.009 | 0.009 | 0.013 | 0.017 | 0.013 | 0.011 | 0.015 | 0.013 | 0.002 |
| UDP-N-acetylmuramoyl-pentapeptide biosynthesis I (meso-diaminopimelate containing) | 0.009 | 0.011 | 0.013 | 0.014 | 0.010 | 0.008 | 0.013 | 0.012 | 0.010 |
| Glycolysis I (from glucose 6-phosphate) | 0.008 | 0.008 | 0.012 | 0.015 | 0.011 | 0.009 | 0.014 | 0.012 | 0.010 |
| S-adenosyl-L-methionine cycle I | 0.008 | 0.010 | 0.013 | 0.015 | 0.010 | 0.009 | 0.013 | 0.012 | 0.008 |
| Peptidoglycan biosynthesis I (meso-diaminopimelate containing) | 0.009 | 0.011 | 0.013 | 0.014 | 0.010 | 0.008 | 0.012 | 0.012 | 0.010 |
| Peptidoglycan biosynthesis III (mycobacteria) | 0.009 | 0.011 | 0.013 | 0.014 | 0.010 | 0.008 | 0.012 | 0.012 | 0.010 |
| Phosphatidylglycerol biosynthesis I (plastidic) | 0.008 | 0.010 | 0.013 | 0.014 | 0.010 | 0.008 | 0.013 | 0.012 | 0.009 |
| Phosphatidylglycerol biosynthesis II (non-plastidic) | 0.008 | 0.010 | 0.013 | 0.014 | 0.010 | 0.008 | 0.013 | 0.012 | 0.009 |
| UDP-N-acetylmuramoyl-pentapeptide biosynthesis II (lysine-containing) | 0.008 | 0.010 | 0.012 | 0.013 | 0.009 | 0.008 | 0.012 | 0.012 | 0.009 |
| L-lysine biosynthesis VI | 0.009 | 0.011 | 0.013 | 0.014 | 0.010 | 0.009 | 0.012 | 0.012 | 0.004 |

Supplemental Table S2. Relative abundance (%) of metabolic pathway of fungal genes in the traditionally fermented dairy products (TFDP) from camel, mare, and cow acquired from phylogenetic investigation of communities by reconstruction of unobserved states 2 (PICRUSt2).

| Metabolic pathway | K1 | K2 | K3 | C1 | C2 | C3 | A1 | A2 | A3 |
| --- | --- | --- | --- | --- | --- | --- | --- | --- | --- |
| Aerobic respiration I (cytochrome c) | 0.079 | 0.056 | 0.066 | 0.086 | 0.094 | 0.089 | 0.088 | 0.088 | 0.087 |
| Aerobic respiration II (cytochrome c) (yeast) | 0.079 | 0.056 | 0.066 | 0.086 | 0.094 | 0.089 | 0.088 | 0.088 | 0.087 |
| Glyoxylate cycle | 0.038 | 0.030 | 0.044 | 0.036 | 0.040 | 0.037 | 0.037 | 0.037 | 0.037 |
| TCA cycle II (plants and fungi) | 0.027 | 0.022 | 0.026 | 0.028 | 0.030 | 0.028 | 0.028 | 0.028 | 0.028 |
| Adenosine ribonucleotides de novo biosynthesis | 0.025 | 0.020 | 0.022 | 0.026 | 0.027 | 0.027 | 0.027 | 0.027 | 0.026 |
| Fatty acid & beta-oxidation (peroxisome, yeast) | 0.024 | 0.035 | 0.034 | 0.021 | 0.021 | 0.020 | 0.020 | 0.020 | 0.021 |
| GDP-mannose biosynthesis | 0.024 | 0.027 | 0.026 | 0.024 | 0.023 | 0.024 | 0.024 | 0.024 | 0.024 |
| tRNA charging | 0.024 | 0.020 | 0.022 | 0.025 | 0.027 | 0.026 | 0.026 | 0.026 | 0.026 |
| Superpathway of adenosine nucleotides de novo biosynthesis I | 0.024 | 0.019 | 0.020 | 0.026 | 0.027 | 0.026 | 0.026 | 0.026 | 0.026 |
| Chitin degradation to ethanol | 0.024 | 0.019 | 0.022 | 0.024 | 0.026 | 0.025 | 0.024 | 0.024 | 0.024 |
| Superpathway of adenosine nucleotides de novo biosynthesis II | 0.022 | 0.018 | 0.019 | 0.024 | 0.025 | 0.024 | 0.024 | 0.024 | 0.024 |
| Pyruvate fermentation to isobutanol (engineered) | 0.023 | 0.022 | 0.023 | 0.021 | 0.021 | 0.021 | 0.021 | 0.021 | 0.022 |
| 4-amino-2-methyl-5-phosphomethylpyrimidine biosynthesis (yeast) | 0.021 | 0.016 | 0.018 | 0.022 | 0.021 | 0.022 | 0.023 | 0.023 | 0.022 |
| Phosphopantothenate biosynthesis I | 0.021 | 0.017 | 0.027 | 0.020 | 0.023 | 0.020 | 0.019 | 0.019 | 0.020 |
| D-myo-inositol (1,4,5)-trisphosphate biosynthesis | 0.020 | 0.019 | 0.021 | 0.020 | 0.022 | 0.020 | 0.020 | 0.020 | 0.020 |
| Glycogen biosynthesis II (from UDP-D-Glucose) | 0.021 | 0.016 | 0.018 | 0.021 | 0.017 | 0.022 | 0.023 | 0.023 | 0.021 |
| Pentose phosphate pathway (non-oxidative branch) | 0.019 | 0.024 | 0.025 | 0.018 | 0.017 | 0.018 | 0.018 | 0.018 | 0.018 |
| Superpathway of L-serine and glycine biosynthesis I | 0.020 | 0.018 | 0.019 | 0.019 | 0.020 | 0.019 | 0.019 | 0.019 | 0.019 |
| Guanosine nucleotides degradation II | 0.020 | 0.028 | 0.028 | 0.016 | 0.000 | 0.016 | 0.017 | 0.017 | 0.016 |
| L-valine biosynthesis | 0.019 | 0.020 | 0.021 | 0.017 | 0.017 | 0.017 | 0.017 | 0.017 | 0.018 |
| Pyrimidine deoxyribonucleotides de novo biosynthesis I | 0.018 | 0.014 | 0.015 | 0.019 | 0.020 | 0.020 | 0.020 | 0.020 | 0.019 |
| Heme biosynthesis I (aerobic) | 0.019 | 0.013 | 0.018 | 0.019 | 0.020 | 0.019 | 0.019 | 0.019 | 0.019 |
| Superpathway of guanosine nucleotides de novo biosynthesis I | 0.018 | 0.015 | 0.015 | 0.019 | 0.020 | 0.019 | 0.019 | 0.019 | 0.019 |
| Superpathway of phosphatidate biosynthesis (yeast) | 0.018 | 0.015 | 0.016 | 0.018 | 0.019 | 0.019 | 0.019 | 0.019 | 0.019 |
| Pentose phosphate pathway | 0.017 | 0.017 | 0.018 | 0.018 | 0.019 | 0.018 | 0.018 | 0.018 | 0.018 |
| 1,3-propanediol biosynthesis (engineered) | 0.017 | 0.014 | 0.016 | 0.018 | 0.020 | 0.019 | 0.018 | 0.018 | 0.018 |
| Superpathway of L-threonine biosynthesis | 0.017 | 0.015 | 0.015 | 0.018 | 0.019 | 0.018 | 0.018 | 0.019 | 0.018 |
| Pyrimidine deoxyribonucleotides biosynthesis from CTP | 0.017 | 0.014 | 0.010 | 0.019 | 0.020 | 0.020 | 0.020 | 0.020 | 0.019 |
| Glycolysis III (from glucose) | 0.017 | 0.015 | 0.015 | 0.018 | 0.018 | 0.018 | 0.018 | 0.018 | 0.018 |
| Superpathway of pyrimidine nucleobases salvage | 0.017 | 0.014 | 0.016 | 0.017 | 0.017 | 0.017 | 0.017 | 0.017 | 0.017 |
| L-proline biosynthesis II (from arginine) | 0.017 | 0.014 | 0.016 | 0.017 | 0.017 | 0.017 | 0.017 | 0.017 | 0.017 |
| Guanosine ribonucleotides de novo biosynthesis | 0.016 | 0.015 | 0.015 | 0.017 | 0.017 | 0.017 | 0.017 | 0.017 | 0.017 |
| Adenine and adenosine salvage III | 0.016 | 0.014 | 0.016 | 0.017 | 0.017 | 0.017 | 0.017 | 0.017 | 0.017 |
| Mevalonate pathway I | 0.016 | 0.013 | 0.015 | 0.017 | 0.017 | 0.017 | 0.017 | 0.017 | 0.017 |
| Superpathway of heme biosynthesis from glycine | 0.016 | 0.013 | 0.008 | 0.018 | 0.019 | 0.018 | 0.018 | 0.018 | 0.018 |
| UDP-N-acetyl-D-glucosamine biosynthesis II | 0.016 | 0.013 | 0.015 | 0.017 | 0.017 | 0.017 | 0.017 | 0.017 | 0.017 |
| Pyrimidine deoxyribonucleotide phosphorylation | 0.015 | 0.013 | 0.013 | 0.017 | 0.017 | 0.017 | 0.017 | 0.017 | 0.017 |
| CDP-diacylglycerol biosynthesis I | 0.015 | 0.012 | 0.013 | 0.016 | 0.017 | 0.017 | 0.017 | 0.017 | 0.017 |
| D-galactose degradation V (Leloir pathway) | 0.016 | 0.016 | 0.017 | 0.016 | 0.000 | 0.016 | 0.017 | 0.017 | 0.016 |
| Urea cycle | 0.015 | 0.013 | 0.008 | 0.017 | 0.017 | 0.017 | 0.017 | 0.017 | 0.017 |
| Formaldehyde assimilation III (dihydroxyacetone cycle) | 0.015 | 0.014 | 0.007 | 0.017 | 0.017 | 0.017 | 0.017 | 0.017 | 0.017 |
| Tetrapyrrole biosynthesis II (from glycine) | 0.014 | 0.014 | 0.006 | 0.017 | 0.017 | 0.017 | 0.017 | 0.017 | 0.017 |
| Sulfate reduction I (assimilatory) | 0.014 | 0.006 | 0.006 | 0.017 | 0.017 | 0.017 | 0.017 | 0.017 | 0.016 |


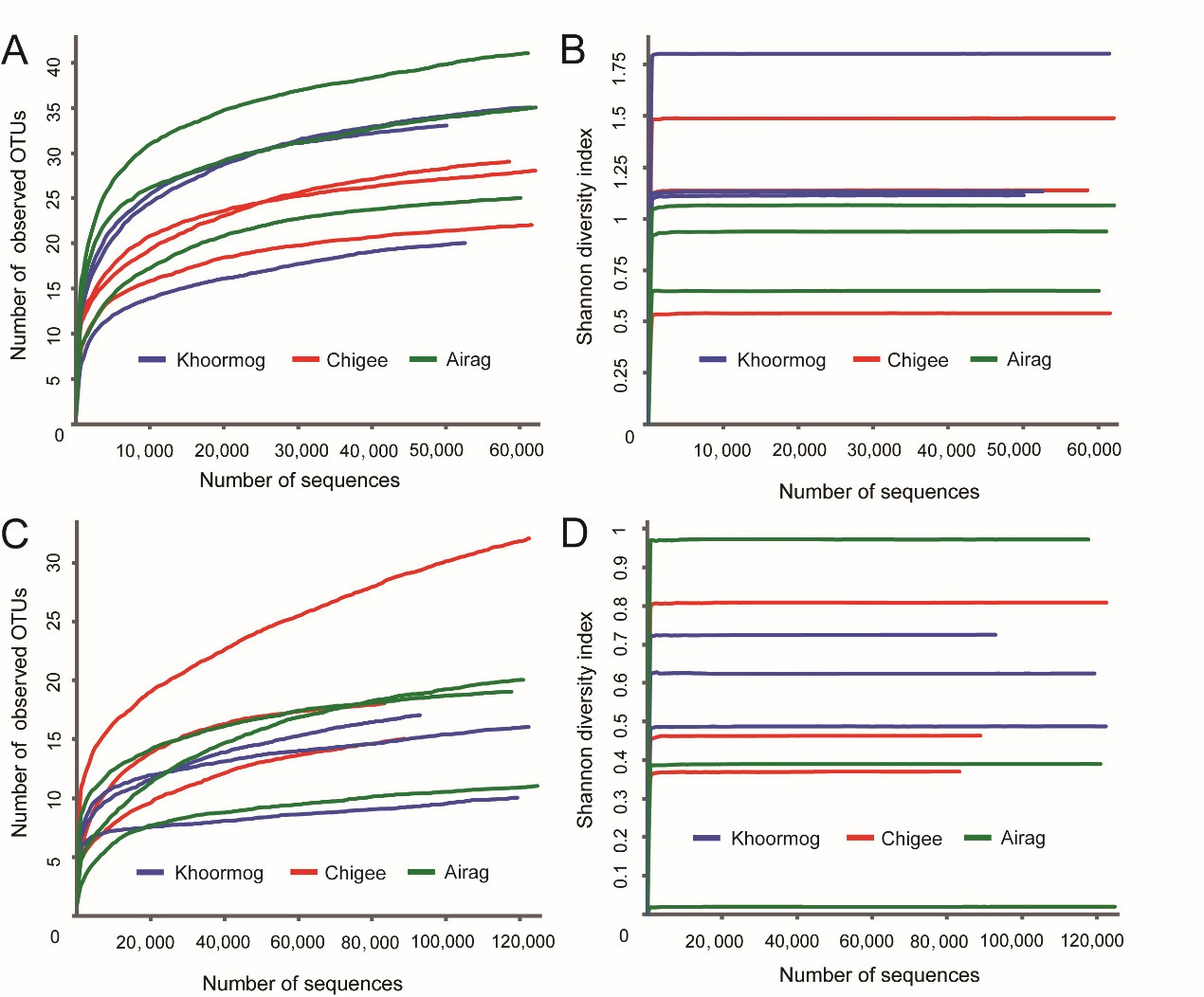


Figure S1. The rarefaction curves (A and C) and the Shannon diversity curves (B and D) for 16S rRNA (A and B) and ITS (C and D) sequencing analyses in Khoormog, Chigee, and Airag. OTUs = operational taxonomic units. The color version is available online.
